# Atlas and Epitome of Diseases of the Mouth, Pharynx and Nose

**Published:** 1903-09

**Authors:** 


**Atlas and Epitome of Diseases of the Mouth, Pharynx and Nose.** By L. GRÜNWALD, M.D. Second Edition. Edited, with additions, by JAMES E. NEWCOMB, M.D. Pp. 219. London: W. B. Saunders & Company. 1903.

We have so recently commended this excellent atlas that little requires to be said beyond reiterating our cordial welcome. It has evidently received such at the hands of the medical profession generally, seeing that within so short a space of time this enlarged and revised edition has been required. All the plates are beautifully drawn and reproduced. Most of them are exceedingly useful and instructive. A few are, we think, unnecessary, inasmuch as they depict conditions with which every practitioner must be familiar. It is, however, not an atlas only. The text is full, clear and accurate, and renders the work as a whole a very serviceable practical text-book.

